# Insights into Hepatic, Neuronal, and hERG Channel Safety of Plant-Derived Active Compounds

**DOI:** 10.3390/jox15060175

**Published:** 2025-10-22

**Authors:** Yosita Kasemnitichok, Sion Lee, Oh Bin Kwon, Tullayakorn Plengsuriyakarn, Kesara Na-Bangchang

**Affiliations:** 1 Graduate Studies, Chulabhorn International College of Medicine, Thammasat University, Pathum Thani 12120, Thailand; yosita.kas@dome.tu.ac.th (Y.K.); tulpleng@tu.ac.th (T.P.); 2 New Drug Development Center, K-MEDI Hub, Daegu 41061, Republic of Korea; sionlee@kmedihub.re.kr (S.L.); kob325@kmedihub.re.kr (O.B.K.); 3 Drug Discovery and Development Center, Office of Advanced Science and Technology, Thammasat University, Pathum Thani 12120, Thailand

**Keywords:** hepatotoxicity, neurotoxicity, cardiotoxicity, plant-derived compounds, hERG patch clamp assay

## Abstract

Curcumin (CUR), atractylodin (ATD), α-mangostin (αMG), ethyl-p-methoxycinnamate (EPMC), ligustilide (LIG), and β-eudesmol (BEU) are commonly used in Thai traditional medicine formulations. This study evaluated the cytotoxic effects of these compounds in HepG2 liver cancer cells and ReNcell VM neural progenitor cells using the resazurin assay, as well as their potential for hERG inhibition in hERG-overexpressing HEK293 cells, utilizing the automated patch-clamp technique. αMG and CUR significantly reduced HepG2 cell viability (IC_50_ = 5.5 and 21 µM, respectively). In undifferentiated ReNcell VM cells, αMG was the most potent inhibitor of cell viability (IC_50_ = 2.1 µM), followed by CUR (IC_50_ = 21.1 µM), while in differentiated ReNcell VM cells, only αMG exhibited significant neurotoxicity (IC_50_ = 6.0 µM). Other compounds showed no significant effects on these cells. ATD, BEU, LIG, and EPMC demonstrated low inhibition of hERG channels (IC_50_ = 26.4, 33.4, 37.3, and 53 µM, respectively), while CUR and αMG displayed weak inhibitory effects (IC50 > 100 µM). αMG may have cytotoxic effects on hepatocytes and neurons at concentrations much higher than when used as medicine or food supplements. At regular clinical doses, αMG, ATD, BEU, EPMC, LIG, and CUR are unlikely to cause significant side effects. However, if these compounds are considered for drug development, their potential effects on hERG channels should be carefully assessed to avoid possible cardiotoxicity. Pharmacokinetics, both preclinical and clinical studies, are necessary to understand the relationship between the plasma concentration profile of EPMC and its potential risks for hepatotoxicity, neurotoxicity, cardiotoxicity, and drug interactions.

## 1. Introduction

**Figure 1:**
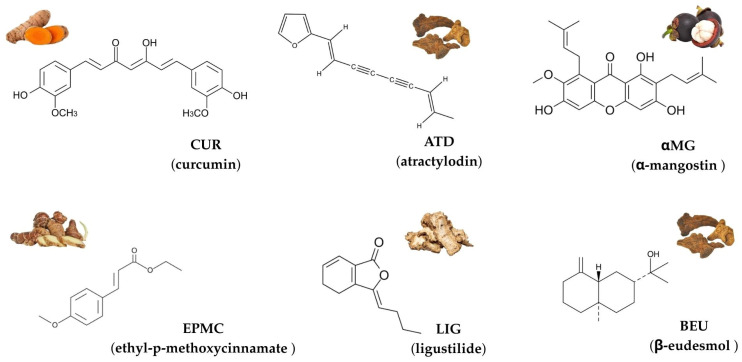
Chemical structures and representative natural sources of six compounds commonly used in Thai traditional medicine: curcumin (CUR), atractylodin (ATD), α-mangostin (αMG), ethyl-p-methoxycinnamate (EPMC), ligustilide (LIG), and β-eudesmol (BEU).

Herbs have gained increasing attention in drug discovery due to their diverse biological activities, including anticancer, anti-inflammatory, and cardioprotective effects [1,2,3]. Among these, curcumin (CUR), atractylodin (ATD), α-mangostin (αMG), ethyl-p-methoxycinnamate (EPMC), ligustilide (LIG), and β-eudesmol (BEU) are commonly used in Thai traditional medicine formulations and stand out for their therapeutic potentials [4,5,6,7,8]. The chemical structures of these six compounds are shown in Figure 1.

CUR, derived from *Curcuma longa*, exhibits anti-inflammatory and anticancer activity [9], while αMG, a xanthone from *Garcinia mangostana*, is recognized for its antioxidant, anti-inflammatory, and antiproliferative effects [10,11,12,13]. ATD and BEU, isolated from *Atractylodes lancea* and EPMC, a derivative of cinnamon essential oils, have demonstrated antimicrobial and anticancer activity [14,15,16]. LIG, found in traditional Chinese herbs such as *Ligusticum* and *Atractylodes*, has been shown for its neuroprotective and anticancer properties [17]. Despite these promising biological activities, limited information exists regarding the cytotoxicity and potential adverse effects of these compounds on human cells. While many natural compounds exhibit beneficial therapeutic effects, their impact on the cardiovascular system is particularly crucial, especially regarding their potential to inhibit the hERG (human ether-a-go-go-related gene) potassium ion (K^+^) channel. The hERG channel plays a crucial role in cardiac repolarization, and its inhibition can lead to QT-interval prolongation in the electrocardiogram, thereby increasing the risk of life-threatening tachyarrhythmias, such as Torsade-de-pointes. Therefore, assessing hERG channel inhibition using hERG-overexpressing HEK293 cells is essential for early-stage safety evaluations of drug candidates to prevent cardiotoxicity [18].

An overview of Thai herbal recipes, including their relevant phytochemicals, ethnomedical origins, and therapeutic indications, is summarized in Table 1.

Given the therapeutic promise of CUR, ATD, αMG, EPMC, LIG, and BEU, it is necessary to investigate their cytotoxicity on liver and neural cells, including their potential cardiotoxic effects through hERG inhibition. This study aimed to evaluate the cytotoxic effects of these compounds in HepG2 liver cancer cells and ReNcell^®^ VM neural progenitor cells using the resazurin assay, along with the hERG toxicity using hERG-overexpressing HEK293 cells through the automated patch-clamp technique [25]. Understanding the cytotoxic and cardiotoxic profiles of these natural compounds will help ensure their continued development as safe and effective therapeutic agents for various diseases.

## 2. Materials and Methods

### 2.1. Chemicals

Atractylodin (ATD), *β*-eudesmol (BEU), and α-mangostin (αMG) were supplied by WAKO (Osaka, Japan). Curcumin (CUR) and ethyl-p-methoxycinnamate (EPMC) were purchased from the Tokyo Chemical Industry (Tokyo, Japan). Ligustilide(LIG), quinidine, acetaminophen and accutase^®^ were supplied by Sigma-Aldrich (St. Louis, MO, USA). The CellTiter-Blue^®^Cell viability assay was purchased from Promega (Madison, WI, USA). Triton X-100 was supplied by Biosesang Inc. (Gyeonggi-do, Republic of Korea). The external standard buffer solution (NaCl and HEPES) and internal standard buffer solution (KF 110 and EGTA) for the hERG inhibition assay were purchased from Nanion Technologies (Munich, Germany).

Stock solutions of all compounds were prepared by dissolving each compound in dimethyl sulfoxide (DMSO) to achieve a concentration of 50 mM. The working solutions were prepared in the relevant media to achieve the desired concentration, with the final DMSO concentration not exceeding 0.2%.

### 2.2. Cell Culture

The HepG2 cell line (Human hepatocellular carcinoma) was obtained from The American Type Culture Collection (Manassas, VA, USA) and maintained in Dulbecco’s Modified Eagle Medium (GIBCO, Carlsbad, CA, USA). The hERG-overexpressing HEK293 cell line was purchased from Eurofins Scientific (Luxembourg, Luxembourg). The cells were cultured in Dulbecco’s Modified Eagle Medium F12 (GIBCO, Carlsbad, CA, USA). All culture media were supplemented with 10% fetal bovine serum (FBS) and 1% penicillin/streptomycin. The ReNcell^®^ VM (immortalized human neural progenitor cells) was purchased from Merck Millipore (Burlington, MA, USA) and was cultured on 60 mm cell culture dishes coated with Matrigel^®^ (Corning Inc., Corning, NY, USA) in ReNcell NSC Maintenance Media (Merck Millipore, Burlington, MA, USA) supplemented with 10% EGF (epidermal growth factor) and 1% penicillin/streptomycin. All cells were incubated at 37 °C in a 5% CO_2_ environment.

### 2.3. Cell Differentiation Assay

ReNcell VM cells were seeded into Matrigel^®^-coated black, clear-bottom 96-well plates at a density of 3.0 × 10^4^ cells per well for differentiation assays [26]. The cells were maintained in ReNcell NSC Maintenance Medium (Merck Millipore, Burlington, MA, USA) without growth factors to induce differentiation. The culture medium was changed every two days for three weeks.

### 2.4. Cell Viability Assay

The effects of the test compounds, namely ATD, BEU, αMG, CUR, EPMC, and LIG, on hepatocellular and neuronal viability were assessed using a resazurin reduction assay [27,28].

#### 2.4.1. Hepatocellular Viability Assay

HepG2 cells (1.5 × 10^4^ cells per 100 µL) were seeded into black, clear-bottom 96-well plates and incubated at 37 °C with 5% CO_2_ for 24 h. The test compounds were prepared in complete media at final concentrations of 0.01, 0.1, 1, 10, and 100 μM and added to each well. For the negative control, 0.2% (*v*/*v*) DMSO was used to represent maximum viability, while 0.01% (*v*/*v*) Triton X-100 was used as the positive control for minimum viability. The plates were incubated at 37 °C under 5% CO_2_. After 20 h of incubation, 20% (*v*/*v*) CellTiter-Blue Cell Resazurin reagent was added to each well, followed by an additional 2–4 h of incubation. Fluorescence intensity, indicating resorufin levels, was measured at 590 nm using a Synergy H1 reader was obtained from Agilent Technologies (Santa Clara, CA, USA). Each data point was normalized based on the maximum and minimum cell viability data and expressed as % Relative viability using the following formula:

$$\%\;Relative\;viability=\frac{\left(Experimental\;data-Minimal\;viability\;data\right)}{\left(Maximal\;viability\;data-Minimal\;viability\;data\right)}\times 100$$

#### 2.4.2. Neuronal Viability Assay

Undifferentiated ReNcell VM cells were seeded into Matrigel^®^-coated black, clear-bottom 96-well plates at densities of 1.5 × 10^4^ per well. The cells were maintained in ReNcell NSC Maintenance Media (Merck Millipore, MA, USA), supplemented with 10% EGF and 1% penicillin/streptomycin, and were incubated at 37 °C with 5% CO_2_ for 24 h.

Differentiated ReNcell VM cells were seeded into Matrigel^®^-coated black, clear-bottom 96-well plates at densities of 3.0 × 10^4^ per well. The ReNcell VM cells were incubated at 37 °C with 5% CO_2_ in the ReNcell Maintenance Media (Merck Millipore, MA, USA) without growth factor. The media was changed every two days for three weeks. Both cell types were treated with test compounds at 0.01, 0.1, 1, 10, and 100 μM. DMSO was used as the control for maximum cell viability, and Triton X-100 was the control for minimum viability. After 20 h of incubation at 37 °C with 5% CO_2_. Cell viability was assessed using the CellTiter-Blue^®^ Cell Resazurin reagent.

#### 2.4.3. hERG Inhibition Assay by Patch Clamping Electrophysiology

hERG channel inhibition assay was performed using patch-clamp electrophysiology. HEK293 cells stably overexpressing hERG channels (Eurofins Scientific, Luxembourg) were cultured in Dulbecco’s Modified Eagle Medium F-12 (GIBCO, Carlsbad, CA, USA) supplemented with 10% (*v*/*v*) fetal bovine serum (FBS) and 1% penicillin/streptomycin at 37 °C with 5% CO_2_ until reaching approximately 80% confluency. Cells were then subcultured using trypsin (HyClone, Marlborough, MA, USA), to enzymatically detach adherent HEK293 cells, centrifuged, and resuspended in the External standard buffer (Nanion Technologies, Munich, Germany).

A gigaohm seal was formed after cell seeding in the NPC-16 chip (Nanion Technologies) wells. Brief suction pulses were applied to achieve whole-cell configuration. The hERG channel activity was assessed using a voltage protocol consisting of steps to −80 mV, −40 mV, +40 mV, −40 mV, and back to −80 mV, triggering tail currents. Each well’s peak hERG tail current served as the baseline activity level. To evaluate the inhibitory effects of the six tested compounds, their stock solutions (50 mM, 5 mM, 0.5 mM, 0.05 mM, and 0.005 mM) were diluted with the external standard buffer to final concentrations of 100 µM, 10 µM, 1 µM, 0.1 µM, and 0.01 µM, and automatically applied to the wells. Subsequently, hERG channel activity was measured again using the same voltage protocol. The relative changes in hERG activity induced by the compounds were calculated and expressed as percentages using the following formula:

% hERG activity = (Peak hERG tail current after compound treatment/Peak hERG tail current before compound treatment) × 100

### 2.5. Statistical Analysis

All statistical analyses were performed using GraphPad Prism version 8.0 software (GraphPad Software Inc., San Diego, CA, USA). Using GraphPad Prism, the IC_50_ (concentration that inhibits cell growth by 50%) values were calculated by non-linear regression analysis. Six independent experiments are represented in the data and are presented as means ± standard error of the mean (SEM). Statistical analyses were conducted using one-way analysis of variance (ANOVA). The statistical significance was set at *p*-value < 0.05.

## 3. Results

### 3.1. Effects of ATD, BEU, EPMC, αMG, CUR, and LIG on HepG2 Cells

**Figure 2:**
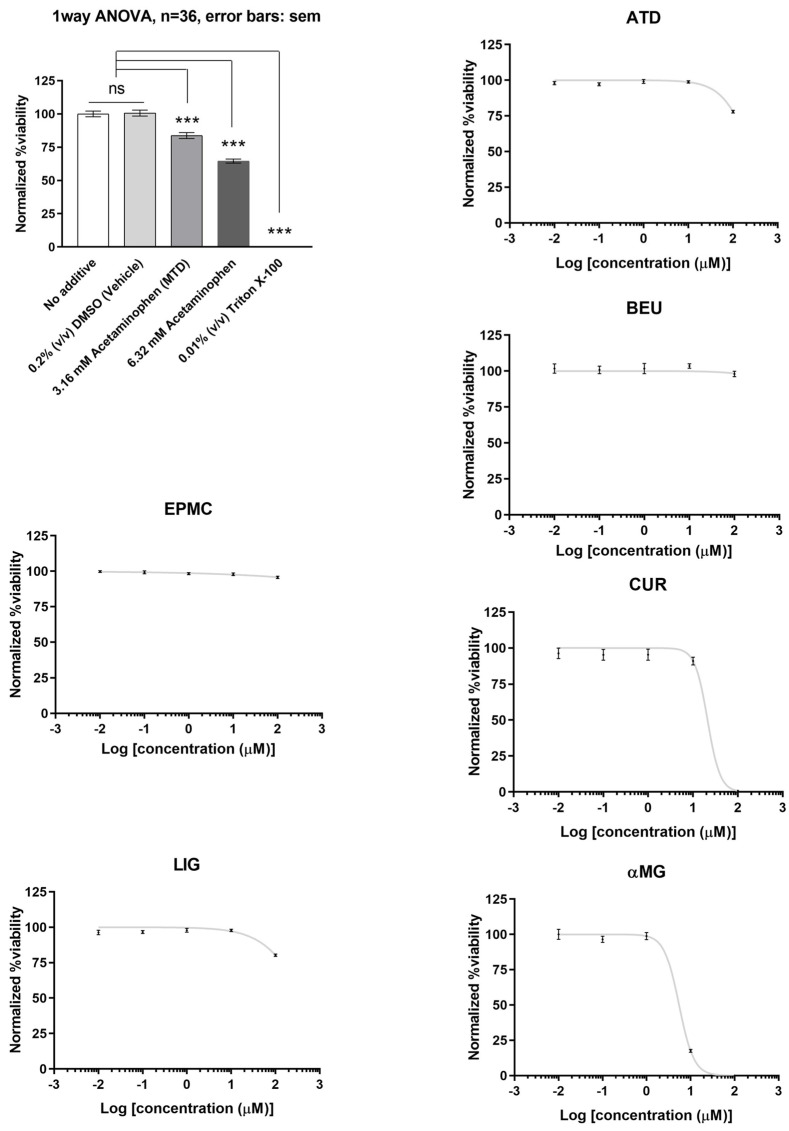
The relative viability of HepG2 cells treated with six compounds at five concentrations (0.01, 0.1, 1, 10, and 100 μM) for 24 h. Maximum cell viability was determined using 0.2% DMSO as a reference for 100% viability, while minimum viability was established with 0.01% Triton X-100 as a reference for 0% viability. IC_50_ values are presented as mean ± SEM from six independent experiments. ns = not significant, *** *p*-value < 0.001.

Of the six compounds, αMG and CUR effectively reduced HepG2 cell viability (IC_50_ = 5.5 µM and 21 µM, respectively). ATD, BEU, EPMC, and LIG did not show significant inhibition of cell viability (IC_50_ > 100 µM). HepG2 cells treated with acetaminophen and triton X-100 (positive control) demonstrated a significant decrease in cell viability when compared to the 0.2% DMSO-treated group (negative control), with a *p*-value of <0.001 (Figure 2).

### 3.2. Effects of ATD, BEU, EPMC, αMG, CUR, and LIG on Neural Progenitor Cells

**Figure 3:**
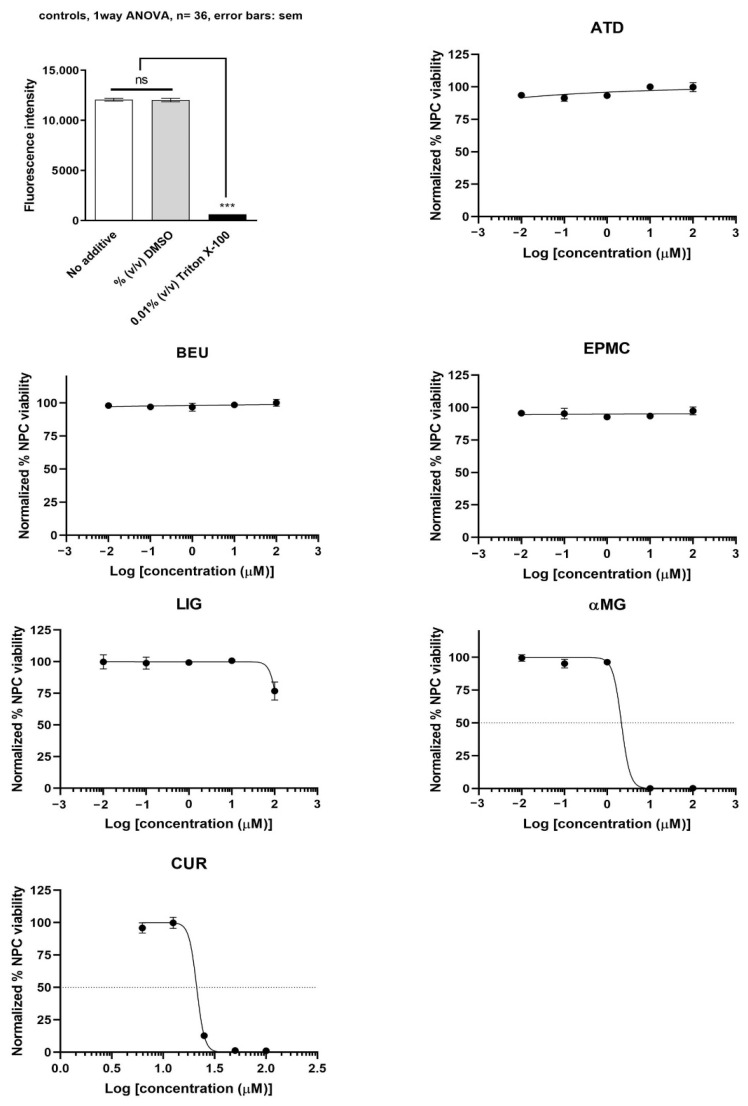
Undifferentiated ReNcell VM cells were treated with six compounds at five concentrations (0.01, 0.1, 1, 10, and 100 μM) for 24 h. Maximum cell viability was determined using 0.2% DMSO as a reference for 100% viability, while minimum viability was established with 0.01% Triton X-100 as a reference for 0% viability. IC_50_ values are presented as mean ± SEM from six independent experiments. ns = not significant, *** *p*-value < 0.001.

αMG showed the most potent inhibition of undifferentiated ReNcell VM cells (IC_50_ = 2.1 µM), followed by CUR (IC_50_ = 21.1 µM). ATD, BEU, EPMC, and LIG did not inhibit undifferentiated ReNcell NSC viability at concentrations up to 100 µM, as compared to Triton X-100 (positive control) (Figure 3).

### 3.3. Effect of ATD, BEU, EPMC, αMG, CUR, and LIG on Differentiated Neuron Cells

**Figure 4:**
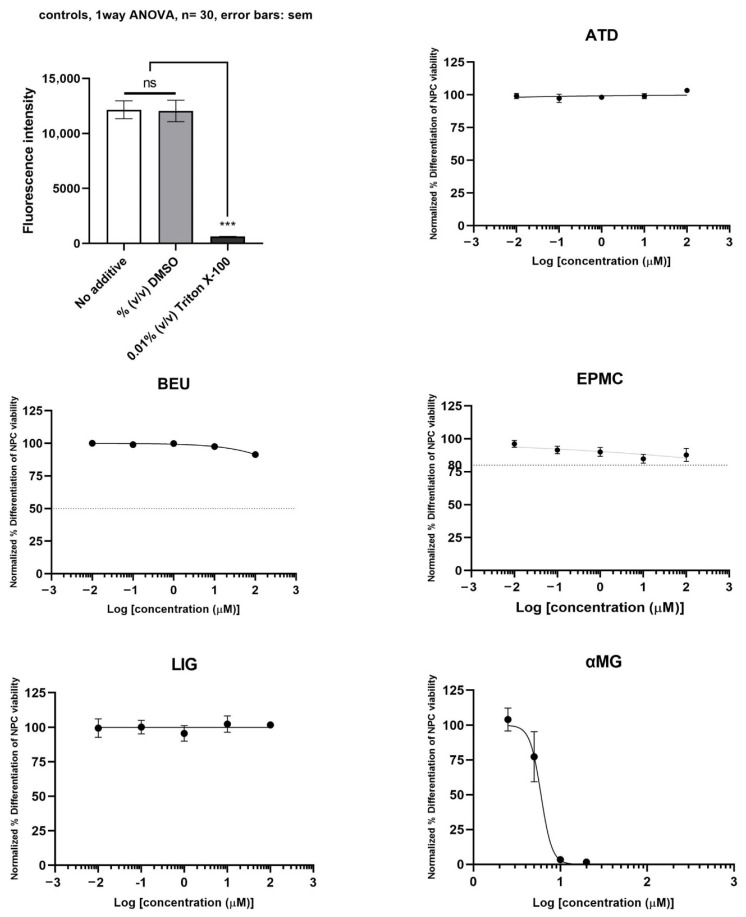
Differentiated ReNcell VM cells were treated with six compounds at five concentrations (0.01, 0.1, 1, 10, and 100 μM) for 24 h. Maximum cell viability was determined using 0.2% DMSO as a reference for 100% viability, while minimum viability was established with 0.01% Triton X-100 as a reference for 0% viability. IC_50_ values are presented as mean ± SE from six independent experiments. ns = not significant, *** *p*-value < 0.001.

The neurotoxicity of differentiated ReNcell VM cells treated with six compounds is shown in Figure 4. αMG exhibited toxicity to differentiated neuron cells (IC_50_ = 6.0 µM). ATD, BEU, EPMC, and LIG did not inhibit the viability of differentiated ReNcell VM cells (IC_50_ >100 µM), compared to Triton X-100 (positive control).

### 3.4. Effects of ATD, BEU, EPMC, αMG, CUR, and LIG on hERG Activity

**Figure 5:**
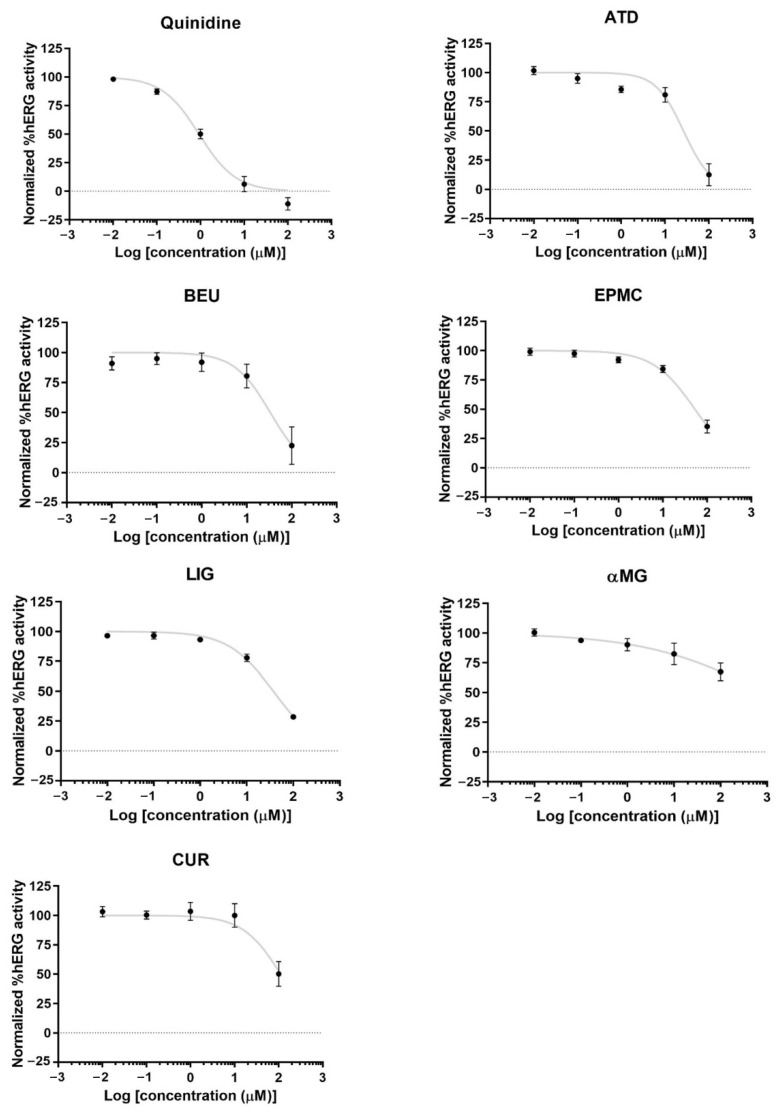
hERG potassium channel activity and concentration-response curves of hERG activity by quinidine (hERG inhibitor) and six compounds in hERG-HEK 293 cells. The result is presented as normalized %hERG activities and means ± standard error (n ≥ 4).

ATD, BEU, LIG, and EPMC exhibited low inhibition of hERG channel activity (IC_50_ = 26.4, 33.4, 37.3, and 53 µM, respectively). CUR and αMG weakly inhibited hERG activity (IC_50_ > 100 µM) (Figure 5).

## 4. Discussion

This research investigated the potential effects of six compounds derived from herbs commonly used in traditional Thai medicine for their effects on hepatocellular viability, neurotoxicity, and cardiotoxicity. *Atractylodes lancea* (AL) has demonstrated significant therapeutic potential, particularly against cholangiocarcinoma (CCA). Atractylodin (ATD) constitutes 14% of AL, has significantly improved delayed gastric emptying, and aids digestion [29]. Additionally, it exhibits antidiarrheal and anti-inflammatory properties by reducing expression of the cytokines IL-1β, IL-6, and TNF-α in the small intestine [10]. β-Eudesmol (BEU), comprising 6.4% of the constituents in AL, displays diverse pharmacological effects, including anti-tumor activity, stimulation of intestinal function, and enhancement of nervous system mechanisms. Both ATD and BEU demonstrated significant cytotoxic activity against cholangiocarcinoma (CCA) cells, with IC_50_ values ranging from 20 to 25 µg/mL. In addition, ATD exhibited inhibitory effects on HepG2 cells (IC_50_ = 26.19 µg/mL) and triple-negative breast cancer (TNBC) cells (IC_50_ = 92.11 µg/mL) [30,31,32]. The capsule formulation of the standardized extract of AL, as well as the capsule formulation of *rikkunshito* containing AL, was developed for further preclinical and clinical studies [33,34]. A phase I clinical trial in healthy Thai and Japanese subjects confirmed the safety of AL for human use, with no adverse reactions or significant changes in hematological or biochemical parameters [35,36]. Pharmacokinetic analysis of AL following a single oral dose of 1000 mg/kg body weight (equivalent to 48.4 mg of ATD) in healthy Thai subjects showed a maximal plasma concentration (C_max_) of 50.35 (13.8–52.90) ng/mL. Similarly, pharmacokinetic analysis of ATD after administration of a single oral dose of rikkunshito (7.5 g/day) in healthy Japanese subjects showed a maximal plasma concentration (C_max_) of 0.002–20 ng/mL ng/mL. However, BEU was reported to block nicotinic acetylcholine receptor channels in mouse skeletal muscles [36,37]. These studies confirm that ATD and BEU exhibited low cytotoxicity towards hepatocellular and neuronal viability, with IC_50_ values exceeding 100 µM. In contrast, both compounds were shown to exhibit cardiotoxicity by inhibiting hERG overexpression in HEK293 cells (IC_50_ = 22.4 µM and 33.4 µM, respectively). Considering the therapeutic plasma concentration of ATD, previous studies have reported that the observed C_max_ was markedly lower than the IC_50_ required to induce cardiotoxicity in vitro [36,38]. This suggests that the risk of cardiotoxic adverse effects from AL is low. Nevertheless, long-term use of AL should be closely monitored, particularly for QT prolongation and potential effects on the nervous system.

Ethyl-p-methoxycinnamate (EPMC) is an active *Kaempferia galanga* (KG) compound. In Thai medical scriptures, KG is critical for various ailments in several traditional formulations, such as Kheaw-hom, Yahom Tultavai and Dephrungsith [7,19,39,40]. EPMC has demonstrated multiple beneficial effects, including anti-inflammatory, antiviral, and anticancer properties, particularly against CCA. The anti-inflammatory properties of EPMC have been shown in several studies. It exhibits potential lipoxygenase (LOX) inhibition in rats’ carrageenan-induced granuloma air pouch and pleurisy models [41]. Additionally, EPMC reduces the expression of cyclooxygenase-2 (COX-2) and NF-kappa-p65 in the oral mucosa of Wistar rats with ulcers [42]. It also downregulates the expression of IL-6 and TNF-α cytokines in HepG2 and A549 cells infected with the dengue virus [43] and decreases the expression of IL-6 and IL-23a in peripheral blood mononuclear cells (PBMCs) from psoriasis patients [19]. Furthermore, EPMC has been shown to inhibit the proliferation of CCA (CL6 cell line) and significantly reduce tumor growth rates in animal models. KG extract at doses up to 5000 mg/kg did not result in death or any significant adverse effects [28]. The potential inhibitory effect of EPMC on the activities of drug-metabolizing enzymes, specifically cytochrome P450 (CYP450), has been investigated both in vitro and in vivo. In the Dephrungsith formulation, EPMC is a major active compound that potently inhibits CYP2D6 in vitro [19], whereas in the Yahom Tultavai formulation, EPMC inhibits CYP1A1, CYP1A2, and CYP2E1 in vivo [39]. The present research demonstrates that EPMC is highly safe for the liver and brain (IC_50_ on cell viability in HepG2 and ReNcell cells > 100 µM). The inhibitory activity of EPMC on the potassium channel in hERG-overexpressing HEK293 cells was relatively weak (IC_50_ = 53 µM). Based on these findings, the cytotoxicity of the compound on hepatocytes, neurons, and cardiomyocytes is relatively low (IC_50_ > 10 µM), suggesting that EPMC could be a promising candidate for further development as a therapeutic agent. Information on the pharmacokinetics, both preclinical and clinical studies, is necessary to understand the relationship between the plasma concentration profile of EPMC and its potential risks for hepatotoxicity, neurotoxicity, cardiotoxicity, and drug interactions.

Ligustilide (LIG) is a bioactive compound found in *Angelica sinensis* (AS), a plant renowned for its medicinal properties, including anti-inflammatory, antioxidant [44], and cardiovascular benefits [45]. Due to these multiple therapeutic effects, the plant is widely used in both traditional Thai and Chinese medicine. Results of the present study revealed no significant inhibitory effect of LIG on HepG2 and ReNcell cells (IC_50_ > 100 µM). This finding aligns with previous studies demonstrating the neuroprotective effects of LIG, including its antidepressant activity mediated through AKT1, MAPK14, and ESR1, as well as signaling pathways such as PI3K/AKT and MAPK [46]. Oral administration of LIG at a 500 mg/kg dose in animals resulted in a plasma C_max_ of 0.66 μg/mL [47]. Our study found that LIG inhibited hERG-overexpressing HEK293 cells with an IC_50_ of 37.3 µM, which is higher than the plasma C_max_ in animals. Nevertheless, close monitoring of adverse effects on cardiovascular and circulatory systems is required to further develop LIG for clinical use. Co-administration with other drugs that inhibit calcium channels should be careful, as LIG has been shown to induce vasodilation by inhibiting calcium channels in mesenteric arteries in animal models [48].

Alpha-mangostin (αMG) is found in high concentrations in the extract of mangosteen pericarp (*Garcinia mangostana*). It has demonstrated various potential health benefits, including antioxidant, cardioprotective, hepatoprotective, and anticancer properties [49]. Previous studies have shown that daily consumption of mangosteen beverages for 30 days significantly increases antioxidant capacity and provides anti-inflammatory benefits without adverse effects on immune, hepatic, or renal functions [50]. Furthermore, αMG has been shown to mitigate doxorubicin toxicity in rats through its antioxidant and anti-inflammatory effects [51], suggesting its cardioprotective properties. In our study, the compound did not exhibit cardiotoxicity in the hERG-overexpressing HEK293 cells (IC_50_ > 100 µM). In contrast, the cytotoxic effects were more pronounced in HepG2, undifferentiated ReNcell, and differentiated ReNcell cells (IC50 values of 2.1, 5.5, and 6.0 µM, respectively). The selective toxic effect of αMG on undifferentiated ReNcell and HepG2 cells compared to differentiated ReNcell cells is likely due to the higher proliferation rates of these undifferentiated cells. Despite low water-solubility, previous studies have shown that αMG reached plasma C_max_ of 1382 nmol/L in rats following an oral dose of 100 mg/kg body weight of αMG [52]. High-dose administration should be done with caution, and brain and liver functions should be closely monitored.

Curcumin (CUR) is a polyphenolic natural compound found in turmeric and has been widely used in Asia and Southeast Asia for centuries. It is commonly incorporated into foods, and recent developments have led to its formulation for therapeutic purposes, such as treating gastric conditions and osteoarthritis [53,54]. Our study found that CUR inhibited the viability of HepG2 cells, ReNcell, and hERG-overexpressing HEK293 cells, with IC_50_ values of 21 µM, 21.16 µM, and 100 µM, respectively. In a pharmacokinetic study, CUR at a dose of 10 g administered to healthy volunteers resulted in a plasma Cmax of 2.3 µg/mL [55], which is lower than the concentrations that induce inhibitory effects on cell viability. However, long-term consumption of CUR or concurrent use with other drugs that may induce hepatotoxicity or neurotoxicity could lead to accumulation in the body, potentially causing toxicity. Therefore, it is essential to further investigate the potential of CUR-drug interactions since CUR is a common dietary supplement in daily life. Taken together, these results indicate that the cytotoxic and cardiotoxic potentials of individual compounds are relatively low at physiologically relevant concentrations. Thai traditional medicine usually uses compounds in polyherbal formulations. We hypothesize that the administration of these bioactive compounds in polyherbal formulations could alter their pharmacokinetic profiles due to natural bioenhancers, thereby enhancing therapeutic efficacy and reducing toxicity.

## 5. Conclusions

In conclusion, at regular clinical doses, αMG, ATD, BEU, EPMC, LIG, and CUR are unlikely to cause significant side effects. However, their impact on hERG channels alerts careful monitoring during drug development to avoid potential cardiotoxic risks. Pharmacokinetics, both preclinical and clinical studies, are necessary to understand the relationship between the plasma concentration profile of EPMC and its potential risks for hepatotoxicity, neurotoxicity, cardiotoxicity, and drug interactions.

## Figures and Tables

**Table 1:** Thai herbal recipes, their relevant phytochemicals, ethnomedical origins, and therapeutic indications.

Herbal Recipe	Phytochemicals	Ethnomedical Origin	Therapeutic Indications	Reference
**Hand and Foot Soaking recipe**	Curcumin (CUR)	Traditional Thai Medicine	Reduce swelling and relieve joint pain	[4]
**Kheaw Hom recipe**	Ethyl p-methoxycinnamate (EPMC)	Traditional Thai Medicine	Treatment of fever	[7]
**Dephrungsith recipe**	Ethyl p-methoxycinnamate (EPMC)	Traditional Thai Medicine	Treatment of psoriasis	[19]
**Thatbunjob recipe**	Atractylodin (ATD, β-Eudesmol (BEU), Ligustilide (LIG), Ethyl p-methoxycinnamate (EPMC)	Traditional Thai Medicine	Treatment of gastrointestinal disorders, including non-infectious diarrhea and indigestion	[20]
**Chantaleela recipe**	Atractylodin (ATD)	Traditional Thai Medicine	Treatment of fever	[21]
**Learng Pid Samud recipe**	Curcumin (CUR)	Traditional Thai Medicine	Treatment of non-infectious diarrhea	[22]
**Prasaprohyai recipe**	Ethyl p-methoxycinnamate (EPMC), Atractylodin (ATD), β-Eudesmol (BEU), Ligustilide (LIG)	Traditional Thai Medicine	Treatment of fever and allergic rhinitis	[23]
**Pericarp of mangosteen fruit**	α-Mangostin (α-MG)	Traditional Thai Medicine	Treatment of diarrhea	[24]

## Data Availability

The original contributions presented in this study are included in the article. Further inquiries can be directed to the corresponding author.
